# Mitral Valve Replacement with Total Chordal Preservation: The Eversion Technique

**DOI:** 10.7759/cureus.15985

**Published:** 2021-06-28

**Authors:** Chandra Prakash Srivastava, Ranajit B Naik

**Affiliations:** 1 Cardiothoracic Surgery, Narayana Multispeciality Hospital, Jaipur, Jaipur, IND

**Keywords:** valve replacement, total chordal preservation, eversion technique, mitral valve stenosis, mitral valve insufficiency

## Abstract

The preservation of the subvalvular apparatus during mitral valve replacement is necessary to maintain the geometry and function of the left ventricle. Although several complete or partial chordal preservation methods have been described, some continue to preserve only the posterior chordal attachments. This is due to a fear of possible patient prosthetic valve mismatch with many well-known chordal preservation techniques. We describe a simple method of preservation of both the leaflets and chordal attachments using a simple eversion technique. In our technique, we retain all the mitral valvular and subvalvular tissue. The excised leaflet ends are everted by the valve sutures to avoid interference with the prosthetic valve leaflets.

## Introduction

Mitral valve repair is the ideal surgical management for a variety of mitral valve pathological states as it provides a competent, non-obstructed valve without compromising the left ventricular function. Despite the advantages of mitral valve repair, many surgeons consider it tedious, time-consuming, and may have high failure rates in inexperienced hands. Thus a large number of patients undergo mitral valve replacement. When repair is not feasible, the preservation of left ventricular function is an important concern.

The effects of the loss of annulo-ventricular continuity and the importance of chordal preservation have been well established and proven by various studies [1, 2] since the introduction of this concept in 1964 by Lillehei and colleagues [3]. A wide variety of techniques of chordal preservation have gained popularity and are now a standard procedure during Mitral Valve replacement [4,5].

Most of the methods described in the literature emphasize the preservation of posterior cusp chords. Despite the anatomical and physiological benefits of the preservation of the subvalvular structures of both valvular leaflets, many surgeons are inclined to preserve only one posterior leaflet. This inclination is due to the technical difficulties in preserving the subvalvular apparatus of both leaflets, which can prolong the operation, the risk of implanting a smaller prosthesis, and the possibility of disrupting the outflow pathway from the left ventricle as well as contact between the prosthesis and subvalvular structures.

With the recent advancements and refinements in techniques and evolution of the low profile bi-leaflet mechanical valves, various methods of total chordal preservation are being described to preserve left ventricular systolic function. The major concern is to avoid the interference of the mechanical prosthetic function by the portions of the retained subvalvular apparatus with reduced risk of left ventricular outflow tract obstruction.

We describe a simple and easily reproducible eversion technique of total chordal preservation during mitral valve replacement, which retains the entire subvalvular apparatus as well as the leaflets without ventricular outflow tract obstruction or interference by retained chordae and leaflet tissue.

## Technical report

Operative technique

**Figure 1:**
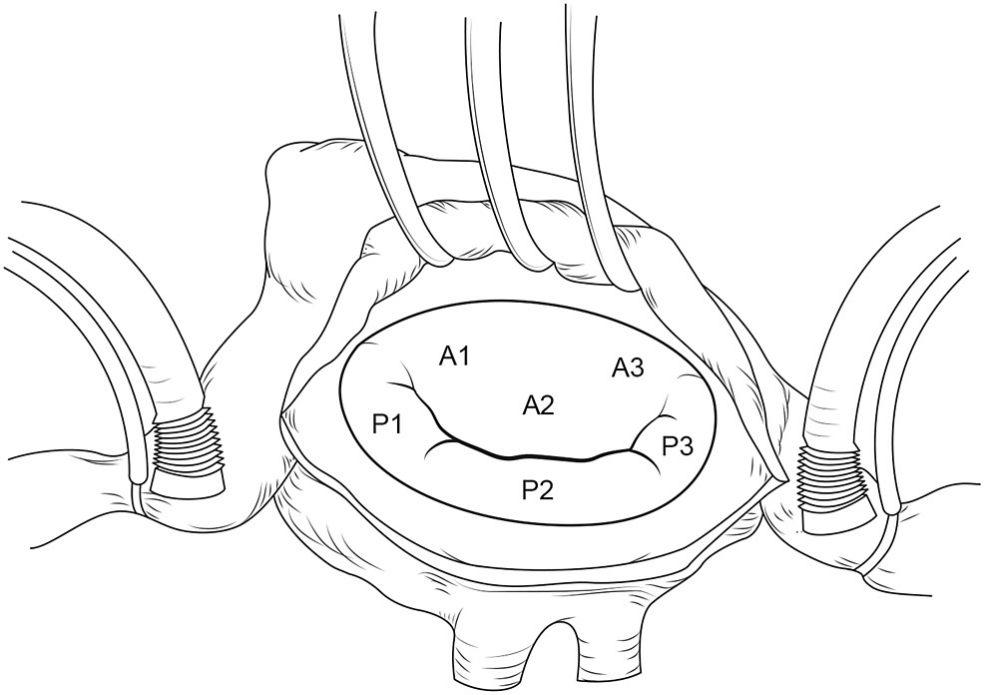
Mitral valve exposed by Left atriotomy

Full median sternotomy is the usual approach to the heart. Cardiopulmonary bypass is established using the standard bicaval cannulation. The aorta is cross clamped and the heart is arrested using antegrade cold blood cardioplegia. The mitral valve is exposed through a classic left atriotomy as shown in Figure 1.

**Figure 2:**
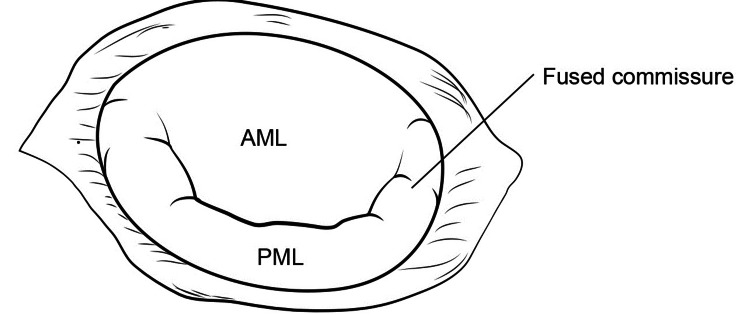
Commissural fusion

**Figure 3:**
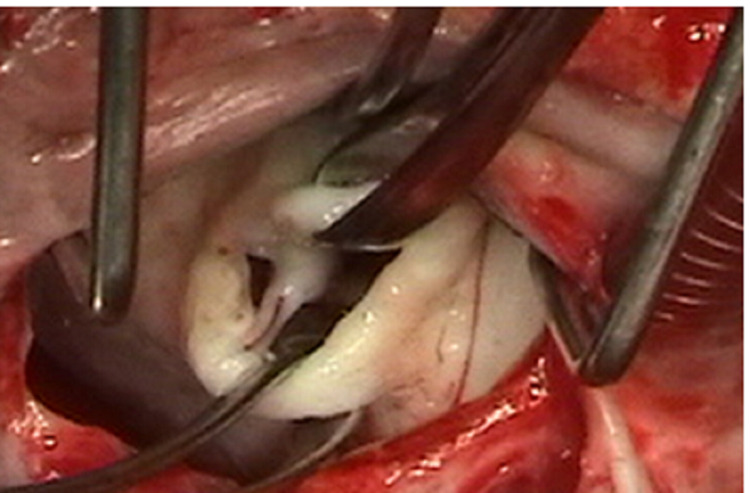
Operative view of commissural fusion

The mitral valve is then examined to determine if there is commissural fusion as noted in Figure 2 and Figure 3.

**Figure 4:**
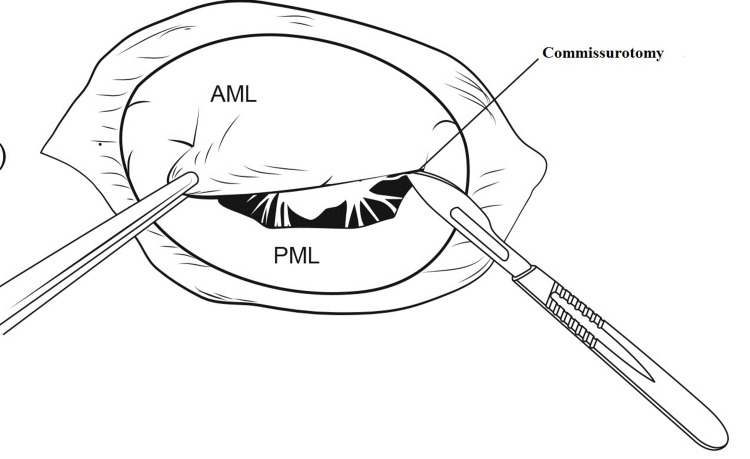
Commissurotomy

Commissurotomy is done as in Figure 4.

**Figure 5:**
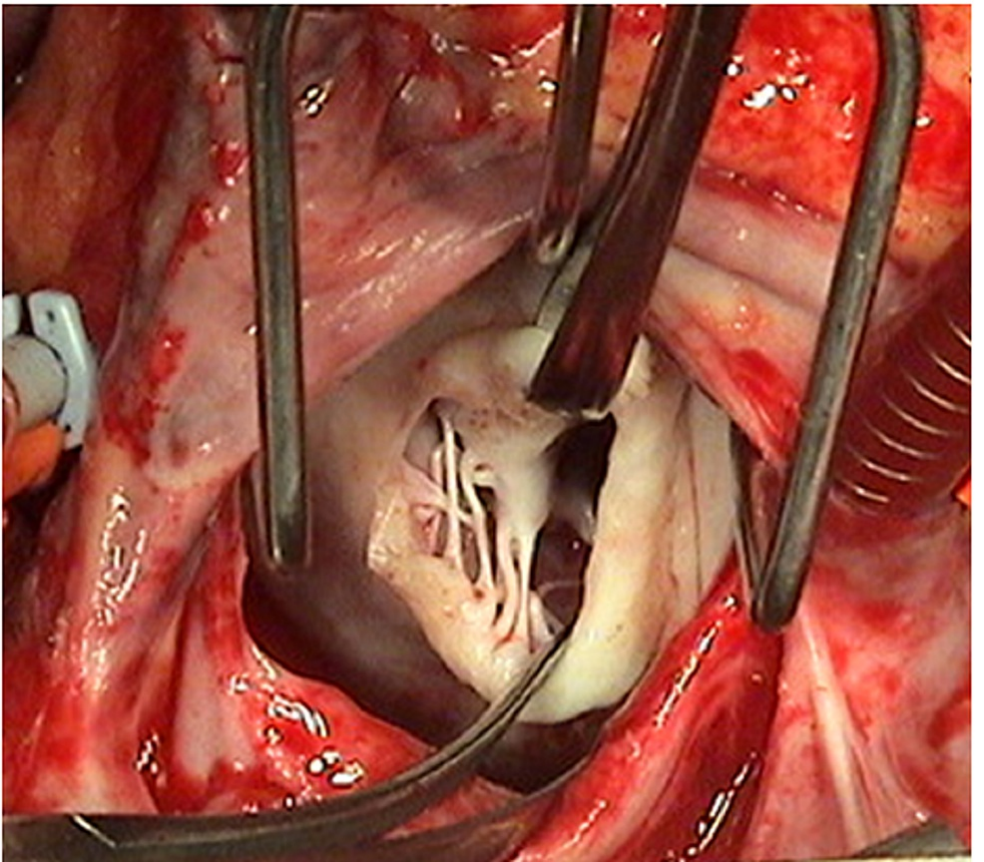
Appearance of the mitral valve post commissurotomy

Post commissurotomy the valve appears as shown in Figure 5.

**Figure 6:**
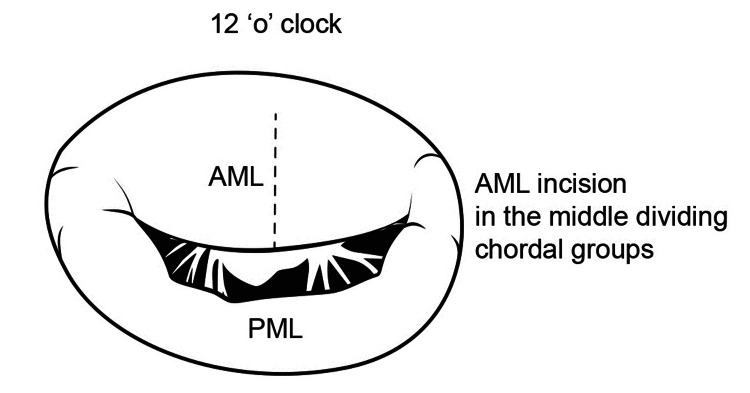
Anterior mitral leaflet slitting

**Figure 7:**
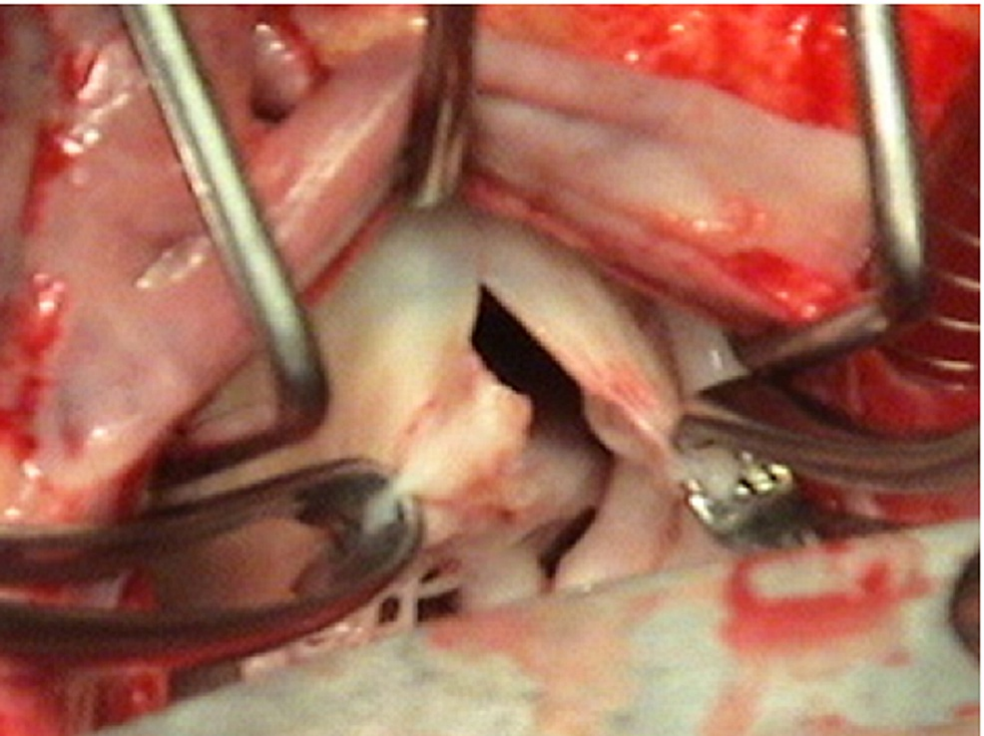
Operative view of Anterior Mitral Leaflet slitting

The anterior mitral valve leaflet (AML) is slit open in the middle between the two main groups of chordae at the 12 o'clock position of the mitral valve when viewed from the operating surgeon's position (Figure 6, 7).

**Figure 8:**
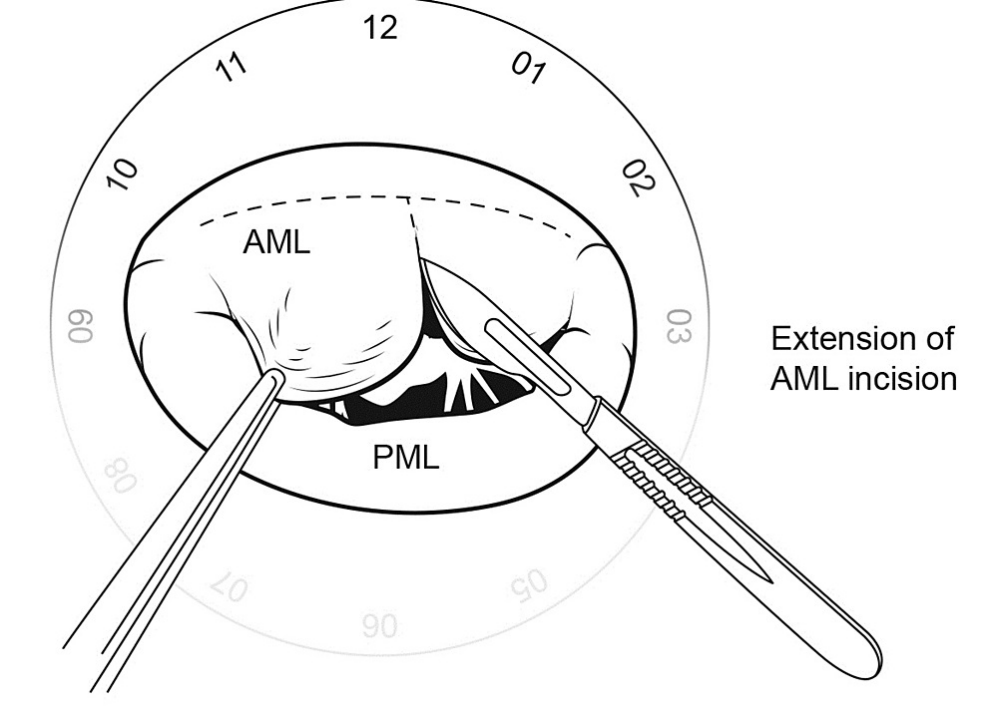
Extension of incision on anterior mitral leaflet

**Figure 9:**
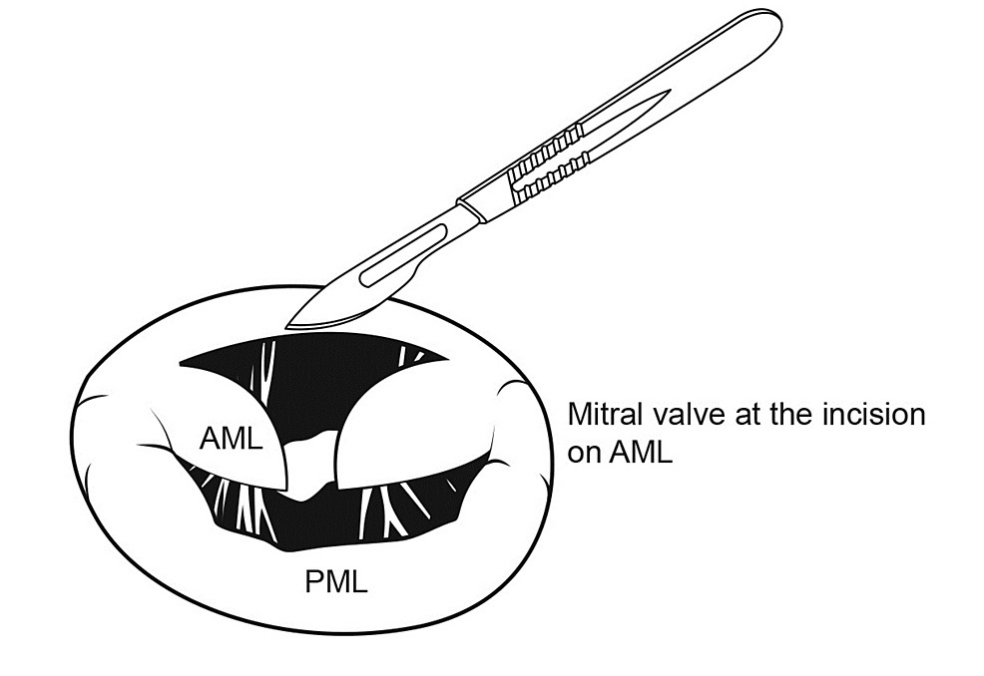
The appearance of the valve on incising the anterior leaflet

The slit incision into the AML is then extended into a T-shaped manner on both sides from the 10 o'clock to the 2 o'clock position, shown in Figures 8, 9.

**Figure 10:**
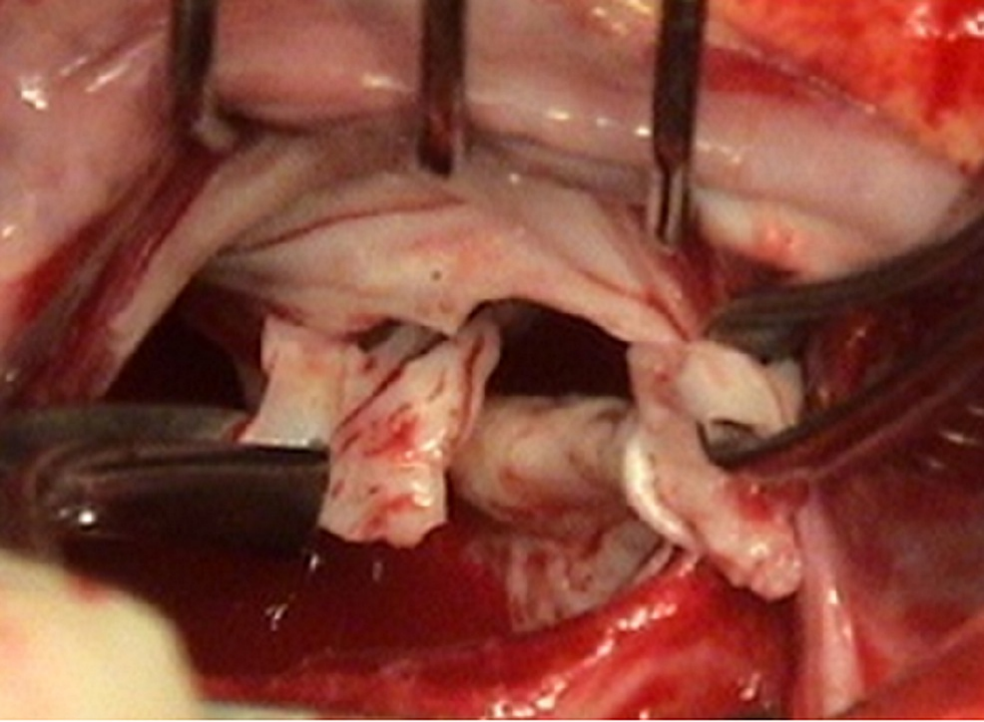
Sagging of the leaflets on AML incision

After incising the AML, as described above, the valve leaflet appears as shown in Figure 10 with two separate leaflet fragments, which assume an appearance of either hanging or sagging.

**Figure 11:**
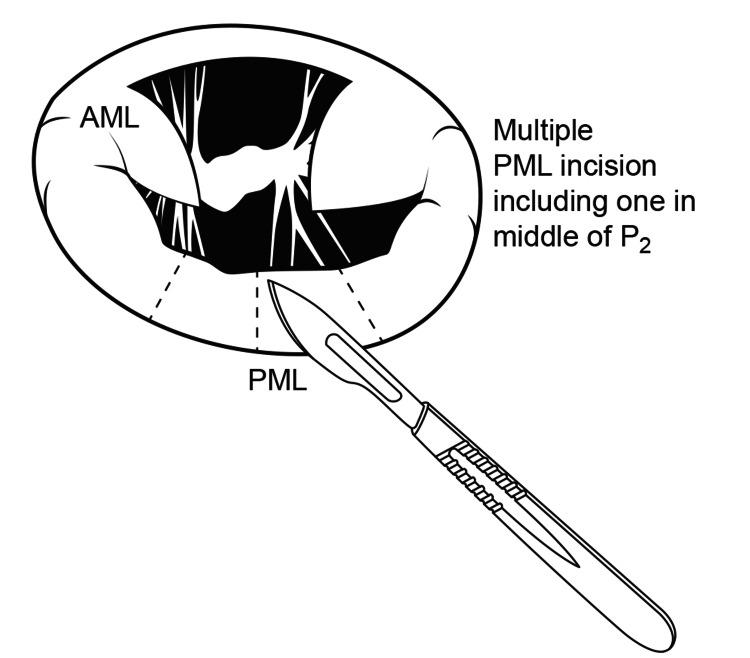
Multiple incisions on Posterior mitral leaflet

Multiple slits are now made into the posterior mitral valve leaflet (PML), starting at the midportion of the P2 segment followed by additional cuts into the P1 and P3 segments, in order to ensure enough space for an adequate or even larger sized prosthetic valve (Figure 11). We must ascertain that the leaflets are supported on both sides by chordae.

**Figure 12:**
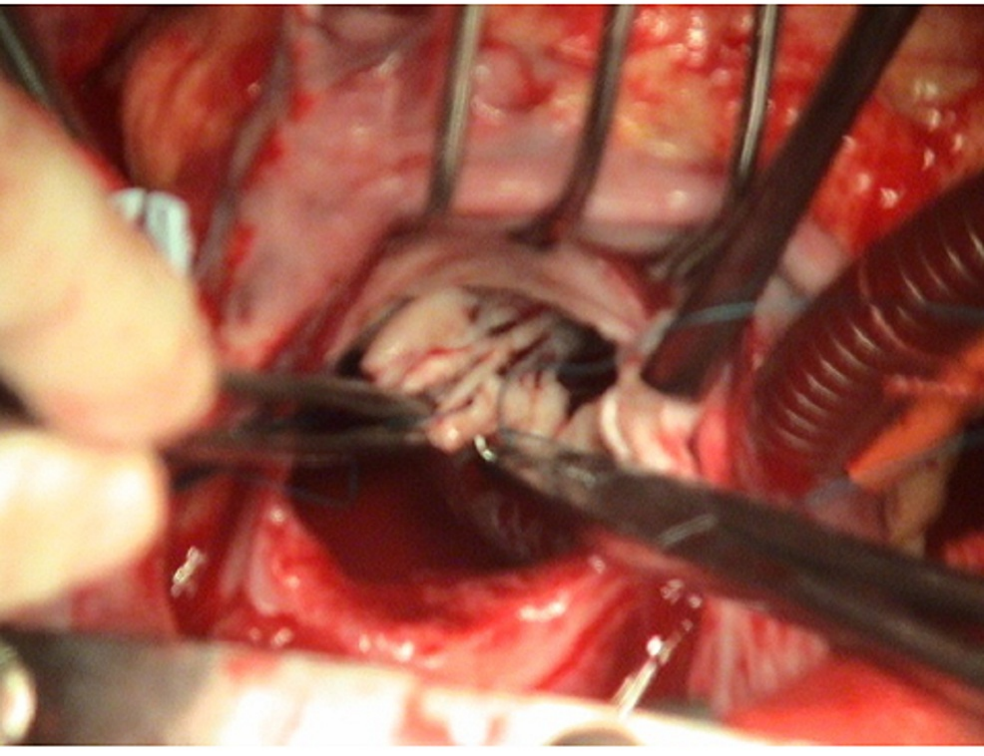
Taking annular sutures sparing chordae

**Figure 13:**
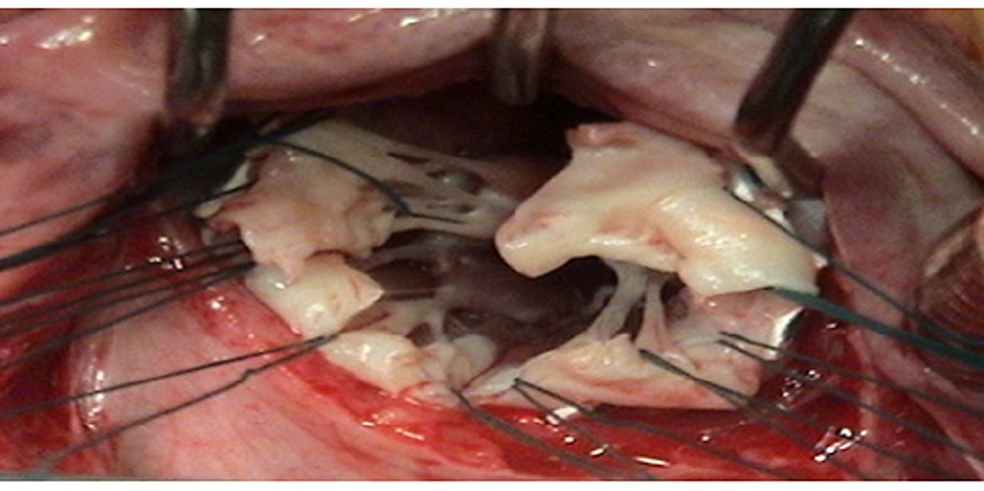
Multiple Annular sutures taken everting the leaflets

Multiple plain interrupted, polyester 2-0 mattress sutures at the base of the remaining leaflets are passed through the annulus and thereafter between the chordae without piercing the chordae (Figure 12, 13).

**Figure 14:**
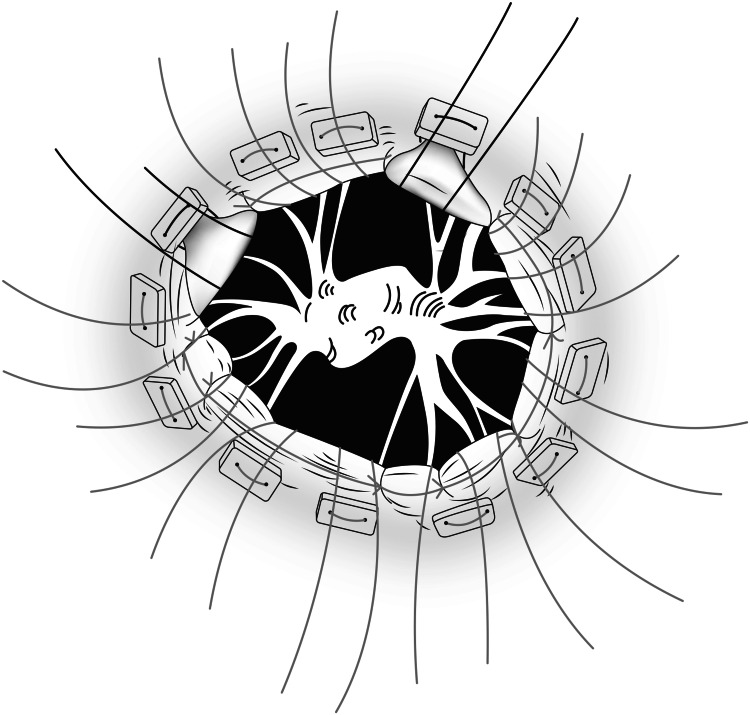
Line diagram depicting Eversion of leaflets

**Figure 15:**
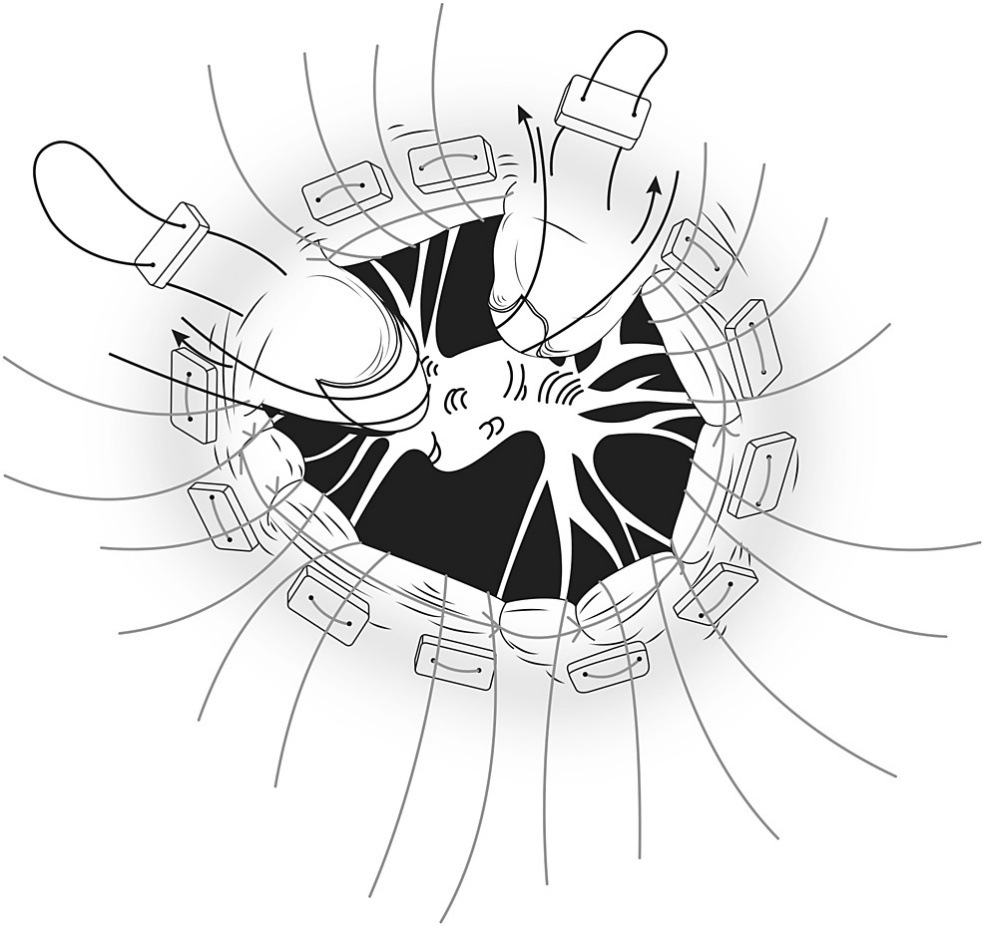
Critical step of leaflet eversion before seating the prosthetic valve

Maintaining the remaining AML and PML leaflets in an everting position as depicted in Figure 14 and Figure 15 will stretch the chordae. The chosen prosthetic valve is seated carefully in the desired position.

**Figure 16:**
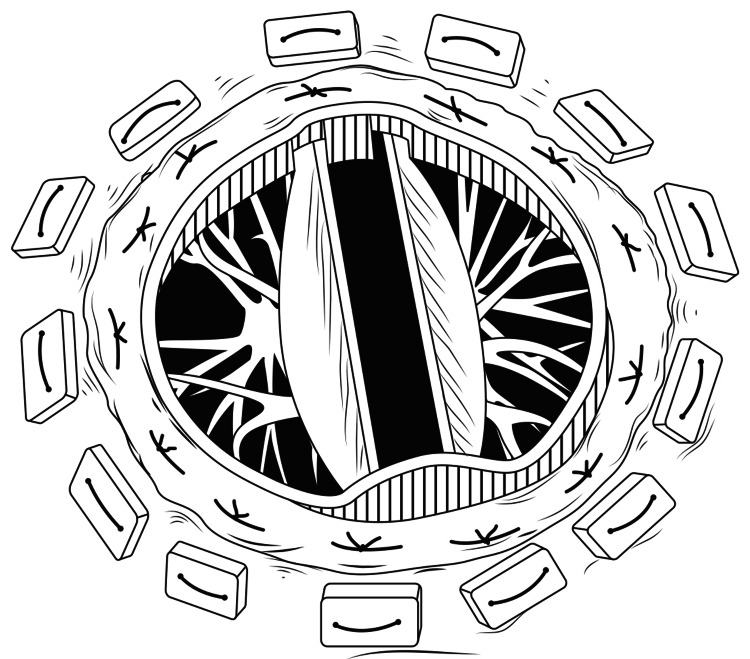
Prosthetic Mitral valve insitu after the procedure by eversion technique

On seating the valve, the chordae automatically become stretched. This allows at least one size larger prosthetic valve to be implanted. The cut portion of the AML is plicated so as to avoid interference with the prosthetic valve's function. The prosthetic valve is seated, as shown in Figure 16.

**Figure 17:**
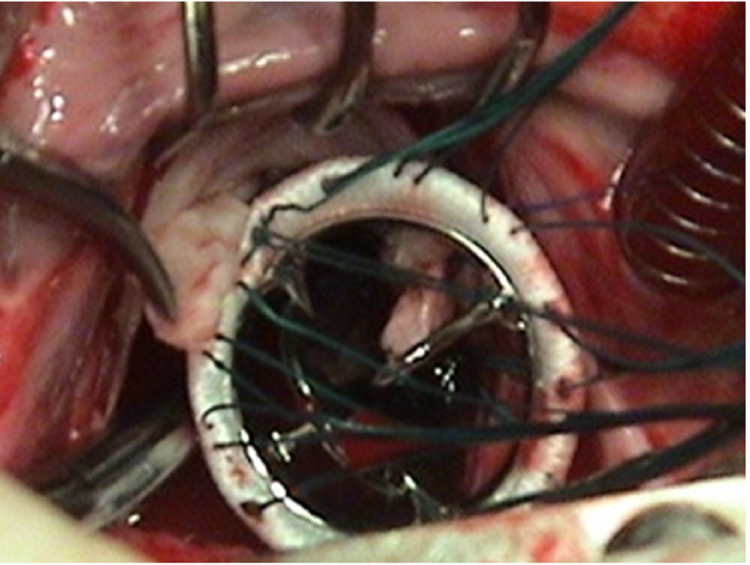
Medtronic Hall valve inserted using Eversion technique

**Figure 18:**
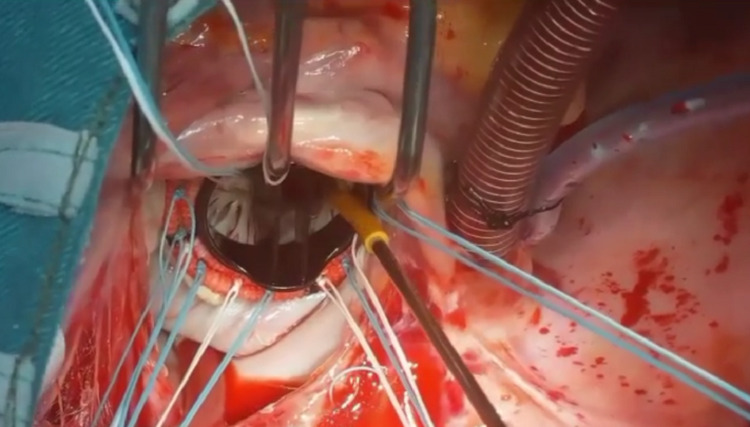
St. Jude Masters Mechanical valve inserted in mitral position using Eversion technique

The choice of the mechanical valve is not affected by the operative technique. We have implanted the Medtronic Hall valve as in Figure 17, St. Jude Masters valve as in Figure 18, and also the TTK Chitra valve in our patients with this eversion surgical technique.

## Discussion

Our eversion surgical technique is simple and easily reproducible. This technique involves T- shaped incision into the AML and multiple incisions into the PML allows for the implantation of an adequate and larger size prosthesis to minimize post-operative patient-prosthesis mismatch. The eversion of leaflets allows the anterior and posterior chordae to fall away from the disc of the prosthesis. We have to be cautious to adjust the tension on the chordae during eversion and avoid too much stress on them. The excessive stretch can lead to chordal rupture and cause entanglement or interfere with the implanted prosthetic valve's function. The method of AML preservation employed in our technique reduces the risk of the systolic anterior motion of the anterior leaflet that could otherwise cause left ventricular outflow tract obstruction. This technique thus ensures the mitral annular-papillary muscle continuity during mitral valve replacement and minimizes the potential risks, complications, and complexities of several other techniques.

## Conclusions

We have adopted this novel technique of total chordal preservation in several patients over the years the implantation of a wide variety of mechanical prosthesis that has shown to be safe in our practice. We hope that this technique being simple and less time-consuming inspires several others to adopt it and ensure better results by total chordal preservation.
